# KiESEL – the children’s nutrition survey module in KiGGS Wave 2

**DOI:** 10.17886/RKI-GBE-2017-106

**Published:** 2017-09-27

**Authors:** Nadine Golsong, Nicole Nowak, Antje Schweter, Oliver Lindtner

**Affiliations:** German Federal Institute for Risk Assessment, Department Exposure, Berlin

**Keywords:** CHILD NUTRITION, EXPOSURE ASSESSMENT, CONSUMPTION DATA, HEALTH MONITORING, KIGGS

## Abstract

Representative food consumption data for children are collected in KiESEL, the German nutrition survey for children aged 6 months up to five years conducted by the German Federal Institute for Risk Assessment (BfR). The data gained will update the consumption data for German children and will fill a data gap that existed for the age group of 5-year-old children. It will provide an actual and comprehensive data basis that will be used for exposure assessment, as part of risk assessment of Germany’s youngest consumers. In the years 2014 to 2017, around 1,000 children will participate in the context of the KiESEL module of the German Health Interview and Examination Survey for Children and Adolescents (KiGGS). During home visits, survey staff conducts a questionnaire-based interview, measures the children’s height and weight and explains the weighing records for the family and the child care workers. The data will be used for risk assessments of the BfR and provided to national and international partners such as the World Health Organization. This article describes the background and objectives of the study as well as its methodology and survey instruments.

## 1. Background and objective

Within the context of consumer health protection, the German Federal Institute for Risk Assessment (BfR) provides scientific risk assessments of substances contained in food. Exposure assessment is part of this risk assessment and analyses the concentration of substances, but also takes into view the population’s dietary habits [1, 2]. Exposure assessment is based on body weight, which often leads to higher estimates for children than for adults. This makes infants and small children a particularly vulnerable population group [3, 4]. The latest nutrition survey for children aged 6 months to 4 years with focus on risk assessment (to evaluate the acute toxicity risks related to pesticide residues) was conducted in 2001/2002 in the context of the VELS study (Verzehrsstudie zur Ermittlung der Lebensmittelaufnahme von Säuglingen und Kleinkindern) [5]. The GRETA study (German Representative Study of Toddler Alimentation) conducted by the Research Institute of Child Nutrition (FKE) also contained representative data on dietary habits of children aged 10 to 36 months [6]. However, changes to dietary recommendations [7], the stream of new food products and changes to dietary habits make it necessary to update the data on children’s food consumption. Between 2014 and 2017, KiESEL (the Children’s Nutrition Survey) will collect data on children’s food intake and update VELS study data. At the same time, KiESEL will seamlessly link with the EsKiMo module (the nutrition module within KiGGS) conducted by the Robert Koch Institute (RKI) [8], which assessed the food intake of children and adolescents aged 6 to 17. In addition, KiESEL is the first study to provide representative data on the dietary habits of five-year-old children [9].

**KiESEL**

The children's nutrition survey to update the consumption data, 2014-2017

**Acronym: KiESEL** - **Ki**nder-**E**rnährungs**s**tudie zur **E**rfassung des **L**ebensmittelverzehr*s*

**Implementation:** German Federal Institute of Risk Assessment (BfR)

**Aim:** Surveying up-to-date and representative data on the food consumption of children aged 6 months to five years. This data provides the BfR with a basis for exposure estimates and improves the assessment of risks related to dietary habits.

**Study design**: Cross-sectional interview survey, anthropometric measures and food diaries

**Population:** Infants and children with permanent residence in Germany

**Sampling:** KiESEL study participants were selected randomly from the cross-sectional sample of KiGGS Wave 2 (registry office sample). An invitation to participate in KiESEL required the prior participation in KiGGS Wave 2.

**Age range:** 6 months to 5 years

**Sample size:** Approximately 1,000 participants

**Survey period:** December 2014-December 2017

More information in German is available at www.bfr.bund.de/de/kiesel-studie.html

Cooperation between the BfR and the RKI will ensure that the data collected in the context of KiESEL can be combined with KiGGS Wave 2 data. The resulting data set merges data on nutrition and health from a representative national sample of children and will provide a basis for further research. An external scientific advisory board [10] coordinates important aspects of the survey and advises the study leaders on methodological questions.

## 2. Methodology

### 2.1 Study design and sampling

The KiESEL sample consists of a partial sampling of the participants from the written questionnaire part of the cross-sectional sample of KiGGS Wave 2 aged of 6 months up to five years, as well as a partial sampling of the physical examinations participants of the cross-sectional sample of KiGGS Wave 2 aged 3 to 5 years.

For the KiGGS Wave 2 study population, 167 representative cities and municipalities (sample points) in Germany were identified. For these sample points a sub-sample stratified by age and sample point was randomly selected based on registry office data. The KiGGS target population and sampling is discussed in detail in the article New data for action. Data collection for KiGGS Wave 2 has been completed in this issue of the Journal of Health Monitoring. Children and adolescents were randomly drawn from the gross sample and assigned to the KiESEL study during sampling for KiGGS Wave 2 and independently of their previous participation in KiGGS. An invitation to participate in KiESEL required prior participation in KiGGS Wave 2. KiESEL aims to collect data of 1,002 children, which amounts to about 83 participants for each age group and gender or 167 children per birth year.

KiGGS Wave 2 participants receive a written invitation to participate in KiESEL. This letter includes the KiESEL flyer as well as a consent form for personal data to be forwarded to the BfR. Following route 15 of KiGGS, the RKI contacts all participants by phone who fail to answer the invitation letter within a given time. Roughly every two weeks the RKI then provides the BfR with the addresses of potential participants who have consented to have their data forwarded. The participants of KiESEL receive a voucher, an age-appropriate toy for their child, and a booklet on child nutrition as an incentive.

The study strictly complies with the German regulations on data protection and has been approved by the Federal Commissioner for Data Protection and Freedom of Information. Participation in the survey was voluntary. Parents and guardians of the children who participated in the survey were informed about the goals and content of the survey as well as about data privacy, and provided their informed consent. The study received a positive vote from the ethics committee of the Berlin Chamber of Physicians (Ärztekammer Berlin, Eth-28/13). Moreover, as part of external quality management measures, KiESEL was audited by aproxima Gesellschaft für Marktund Sozialforschung Weimar mbh.

### 2.2 Assessment methods and testing instruments

**Figure 1:**
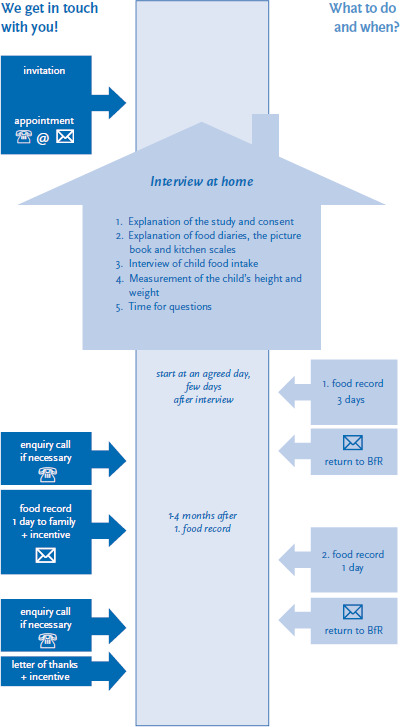
KiESEL study stages Source: Modified according to [9]

KiESEL’s methodology is based on the protocols of the EsKiMo and VELS studies [5, 8, 11]. The KiESEL study team invites families to participate in the survey, contacting them either by phone, via email or post letter, and arranges an appointment with the respective sample point for a home visit (Figure 1).

Appointments take place at the families’ house or in a survey vehicle and require about one hour.

#### Family weighing record

**Figure 2:**
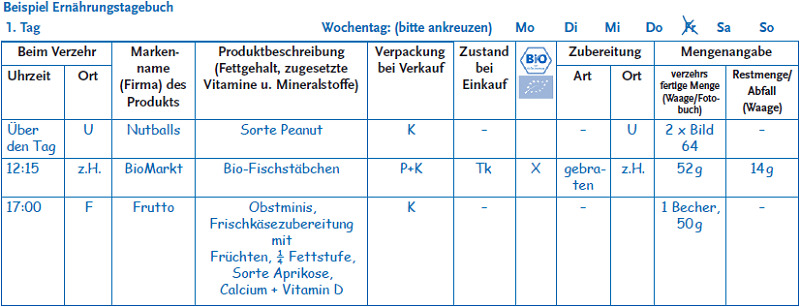
Sample page of a weighing record Source: Own figure (in German)

Families record their child’s food intake through a weighing record for three consecutive days followed later by an additional day. This allows short and long term exposure assessment [12, 13]. To ensure that these measurements are independent of each other, the single-day weighing record is scheduled to be recorded at least two weeks after the 3-day weighing record. The maximum time allowed between the two records is four to eight weeks, for infants and three to four months for elder children. To support the evaluation of risks related to the consumed amounts of food, families are asked to record in detail the amounts of food and beverages consumed (Figure 2).

To record the really consumed amounts, the survey requires participants to weigh the actual amount of food on the plate and, where applicable, leftovers as well. Besides providing information on the foods eaten, participants are also asked to provide the recipes of self-cooked meals. To weigh food, respondents are given kitchen scales. They are also required to record out-of-home consumption (such as ice-cream, snacks or fast food), the amounts of which they then estimate based on amounts as labelled on the food packaging or a picture book provided by KiESEL.

#### Estimate records for child day care facilities

A complete picture of child nutrition also includes the food consumed during the time spend in child day care. A large number of children in Germany is cared for outside their home, for example in kindergartens. According to the Federal Statistical Office this applies to around 32.7% of 0-to 2-year-olds and 93.6% of 3-to 5-year-olds [14]. In parallel with the food records kept at home of the study participants, these institutions provide a food intake estimate for three consecutive days and an additional, unrelated day. The food record for day care workers was adapted after pre-tests in the context of the KiESEL study and now requires less detailed descriptions of food and meals.

Families and day care workers are given the KiESEL picture book to help them to estimate the amounts of food a child consumes either outside their home or in day care facilities. The book contains pictures of children’s portions of different foods in varying portion sizes. Participants can also use household measures and the amounts printed on food packaging. The picture book was developed specifically for KiESEL and its specific age group. It contains 65 picture series as well as individual pictures which were provided by the FKE, the International Agency for Research on Cancer (IARC), the Max Rubner Institute (MRI) and the Pilot study for the Assessment of Nutrient intake and food Consumption Among Kids in Europe (PANCAKE), as well as a set of silhouettes of different foods developed by the BfR. Participants were asked to only use the picture book if they were un able to judge amounts based on the weight indications on food packaging or weigh out-of-home foods [13].

**Info box: F ocuses of the KiESEL questionnaire**

**General information**

Gender

Month and year of birth

Year of birth of parents

**Current dietary habits**

Special diets

Use of salt and oils

Dietary supplements

Consumption of raw foods

**Diet during first year**

Breastfeeding

Infant formula

Solid foods

**Out-of-home consumption**

Meals and food in child care facilities

**Food propensity questionnaire**

Baby food

Cereals and special food for children

Milk and milk substitute products

Meat and sausages

Offal

Fish and seafood

Tea

**Attitudes towards nutrition**

Food Additives

Genetic engineering

Organic food

Food record data are entered into a version of EATv3, a software that was adapted for the needs of KiESEL and developed in 2001 for the VELS study at the University of Paderborn [5].

#### Interview on food intake

The interview on child food intake is based on a questionnaire that standardises data collection on the child, dietary habits and seldom eaten foods (Info box). It supplements the food records and broadens the scope of the details already collected in the KiGGS Wave 2 questionnaire. Following the appointment, questionnaire data are stored in LimeSurvey, a web-based application.

#### Recording body weight and height

Reliable values for the weight and height of children require standardised measurements [15]. Height in these age groups is measured using a mobile measuring board (seca 417, Hamburg, measurement accuracy: ± 0.5cm) with infants lying down. Weight is measured with mobile and calibrated baby scales (seca 336, Hamburg, measurement accuracy: up to a weight of 5kg: ± 0.005kg; for 5-15kg: ± 0.01kg). For children already able to stand upright, height is measured standing with a mobile stadiometer (seca 217, Hamburg, measurement accuracy: ± 0.5cm). They are weighed using mobile and calibrated scales (seca 877, Hamburg, measurement accuracy: ± 0.1kg).

## 3. Outlook

One of the BfR’s central tasks is to scientifically assess the risks related to food, feed as well as substances and products, and to use this data to enhance the German government’s consumer health protection efforts. The collected data on dietary habits of children allow an estimation of the daily mean and high intake levels of contaminants [16-19], pesticide residues [20] and food additives [21] through food. These estimates are required in food safety evaluations and also to define the maximum limits for particular substances in food [9]. Beyond this, the KiESEL study also collects up-to-date data on child nutrient intake in Germany. These data sets serve to describe the nutritional status of infants, toddlers and children [22] and provide policy-makers with a scientific basis for their decisions. To help implement risk assessments at the level of the European Union and worldwide, the data are made available to the Comprehensive European Food Consumption Database of the European Food Safety Authority (EFSA) and the World Health Organization (WHO) [23, 24].

KiESEL is the first study to collect basic data on food consumption in child day care facilities and families as a basis for exposure assessment by age group [25]. Due to the high number of children in child day care, data from these facilities is essential to get a total dietary exposure of the children. Overall, the compliance of the child day care workers to participate in the survey was good. The field study phase will be completed by the end of 2017. Data processing will begin afterwards, and the recorded food will be categorised using the codes of the German Nutrient Database and FoodEx2, a standardised system developed by the EFSA to classify and describe food for exposure assessment. Initial results are expected for 2018. Moreover, a tender is being considered for further research to disaggregate food intakes into categories as defined in the regulation for pesticide residues.

**KiGGS Wave 2**

Second follow-up to the German Health Interview and Examination Survey for Children and Adolescents

**Data owner:** Robert Koch Institute

**Aim:** Providing reliable information on health status, health-related behaviour, living conditions, protective and risk factors, and health care among children, adolescents and young adults living in Germany, with the possibility of trend and longitudinal analyses.

**Study design**: Combined cross-sectional and cohort study conducted as an examination and interview survey

**KiGGS cross-sec tional study**

**Population:** Children and adolescents with permanent residence in Germany

**Sampling:** Samples from official residency registries - randomly selected children and adolescents from the 167 cities and municipalities covered by the KiGGS baseline study

**Age range:** 0-17 years

**Sample size:** Approximately 15,000 participants

**KiGGS cohort study**

**Sampling:** Re-invitation of everyone who took part in the KiGGS baseline study (2003-2006; aged between 0 and 17 at that time) and who was willing to participate in a follow-up

**Age range:** 10-29 years

**Sample size:** Approximately 10,000 follow-up participants

**Survey period:** September 2014-August 2017

**Modules:** BELLA, EsKiMo, GerES, KiESEL, MoMo

More information is available at www.kiggs-studie.de/english

## Key statements

- KiESEL provides up-to-date consumption data on the dietary habits of children aged 6 months to 5 years.
- KiESEL will update the representative consumption data for German children and will fill the data gap for the age group of 5-year-old children.
- The comprehensive data will be used for exposure assessment, as part of BfR risk assessment of Germany’s youngest consumers.
- Data enhances food safety, consumer and child health protection.
